# Prenatal Diagnosis of Fragile X Syndrome in a Twin Pregnancy Complicated by a Complete Retraction

**DOI:** 10.3390/genes9060287

**Published:** 2018-06-07

**Authors:** Yael Prawer, Matthew Hunter, Sara Cronin, Ling Ling, Solange Aliaga Vera, Michael Fahey, Nikki Gelfand, Ralph Oertel, Essra Bartlett, David Francis, David Godler

**Affiliations:** 1 Monash Genetics, Monash Health, Clayton, 3168 Victoria, Australia; matthew.hunter@monashhealth.org (M.H.); michael.fahey@monashhealth.org (M.F.); nikki.gelfand@monashhealth.org (N.G.); 2 Department of Paediatrics, Monash University, Clayton, 3168 Victoria, Australia; 3 Cyto-Molecular Diagnostic Research Laboratory, Victorian Clinical Genetics Services and Murdoch Childrens Research Institute, Royal Children’s Hospital, Parkville, 3052 Victoria, Australia; sara.cronin@vcgs.org.au (S.C.); ling.ling@mcri.edu.au (L.L.); solange.aliagavera@mcri.edu.au (S.A.V.); ralph.oertel@vcgs.org.au (R.O.); essra.bartlett@vcgs.org.au (E.B.); david.francis@vcgs.org.au (D.F.); david.godler@mcri.edu.au (D.G.); 4 Dentistry and Health Sciences, Department of Paediatrics, Faculty of Medicine, University of Melbourne, Parkville, 3052 Victoria, Australia

**Keywords:** fragile-X syndrome, *FMR1* gene, methylation, mosaicism, expansion, retraction

## Abstract

Fragile X syndrome (FXS) is usually associated with a CGG repeat expansion >200 repeats within the *FMR1* gene, known as a full mutation (FM). FM alleles produce abnormal methylation of the *FMR1* promoter with reduction or silencing of *FMR1* gene expression. Furthermore, premutation (PM: 55–199 CGGs) and full mutation alleles usually expand in size when maternally transmitted to progeny. This study describes a PM allele carried by the mother decreasing to a normal sized allele in a male from a dichorionic diamniotic (DCDA) twin pregnancy, with the female twin inheriting FM (200–790 CGGs), PM (130 CGGs) and normal-sized (39 CGGs) alleles. Further evidence of instability of the maternal PM allele was shown by a male proband (older brother) mosaic for PM (CGG 78 and 150 CGGs) and FM (200–813 CGGs), and a high level of *FMR1* promoter methylation, between 50 and 70%, in multiple tissues. The fully-retracted, normal-sized allele was identified by PCR CGG sizing in the male twin, with no evidence of a FM allele identified using Southern blot analysis in multiple tissues collected postnatally and prenatally. Consistent with this, prenatal PCR sizing (35 CGGs) showed inconsistent inheritance of the maternal normal allele (30 CGGs), with single-nucleotide polymorphism (SNP) linkage analysis confirming that the abnormal *FMR1* chromosome had been inherited from the mother’s PM chromosome. Importantly, the male twin showed no significant hypermethylation of the *FMR1* promoter in all pre and postnatal tissues tested, as well as normal levels of *FMR1* mRNA in blood. In summary, this report demonstrates the first postnatal follow up of a prenatal case in which *FMR1* mRNA levels were approaching normal, with normal levels of *FMR1* promoter methylation and normal CGG size in multiple pre and postnatally collected tissues.

## 1. Introduction

Fragile X syndrome (FXS) is a common single gene cause of intellectual disability and co-morbid autism with a frequency in the general population of 1 in 4000 males and 1 in 8000 females [1]. FXS is usually associated with the expansion of a CGG repeat sequence within the *FMR1* promoter to ≥200 CGG repeats, known as a full mutation allele (FM). FM alleles are usually associated with epigenetic changes within the *FMR1* promoter that are not permissive of transcription, including increased DNA methylation [2]. The resulting decrease in *FMR1* mRNA levels and loss of the *FMR1* protein (FMRP) are thought to be the primary causes of the neurodevelopmental phenotype observed in FXS [3]. Smaller *FMR1* allele classes include normal range (<45 CGGs), grey zone (45–54 CGGs) and premutation (PM) (55–199 CGGs) alleles and have an unmethylated *FMR1* promoter that is permissive of transcription and FMRP expression. PM alleles have been primarily associated with adult onset disorders, including Fragile X-Associated Primary Ovarian Insufficiency (FXPOI) and Fragile X Tremor-Ataxia Syndrome (FXTAS) [4].

Importantly, PM and FM alleles usually expand in size in progeny when they are maternally transmitted. Figures regarding the risk of expansion, based on the size of the maternal allele, are used to counsel couples consenting for prenatal diagnosis of the condition [5,6]. Conventionally, prenatal testing involves fetal sex determination by karyotype/FISH, followed by sizing of the *FMR1* allele either through Southern blot analysis and CGG PCR sizing or through triplet repeat primed PCR and linkage techniques [7]. Here, we present an important case with potential implications for counselling following FXS prenatal testing, where a complete retraction or deletion to a normal size allele was identified in a twin pregnancy of a PM mother. This PM mother also had an older child affected with FXS who was mosaic for both PM and FM alleles.

## 2. Methods 

### 2.1. Ethics

The follow-up clinical and molecular assessments consequent to initial genetic counseling were conducted as part of the FREE FX study, and were approved by the Royal Children’s Hospital Research and Ethics Committee (HREC 33066 A); Approved 29 November 2016).

### 2.2. Sample Processing

Chorionic villus (CVS), amniotic fluid, and umbilical cord samples were collected and processed as per standard protocols for molecular diagnostic testing at the Victorian Clinical Genetics Services (VCGS). Venous blood samples of 3–10 mL were collected in ethylenediaminetetraacetic acid (EDTA)-treated tubes. Two milliliter saliva samples were collected using the Oragene^®^ DNA Self-Collection Kit (DNA Genotek Inc., Ottawa, ON, Canada) and isolated as per the manufacturer’s instructions. Up to four buccal epithelial cell samples were collected per participant using the Master Amp Buccal Swab Brush Kit (Epicentre Technologies, Madison, WI, USA). Each swab was inspected independently for blood contamination by at least two staff members at the time of sample collection, and/or at the time of sample receipt prior to processing. Brushes that had confirmed blood contamination were discarded. DNA was extracted from the processed prenatal and postnatal samples using the NucleoSpin^®^ Tissue genomic DNA extraction kit (Machery-Nagel, Duren, Germany).

### 2.3. CGG Sizing

First line *FMR1* testing was performed on blood or saliva DNA at the VCGS. This was conducted using a fully validated PCR amplification assay with a precision level of ±1 repeat and a limit of detection of 170 CGG repeats in males and 130 CGG repeats in females [8]. Second line confirmatory testing involved the identification of repeat sequences in the PM range through Southern blot analysis, performed on samples with inconclusive 1st line PCR results due to either ‘one peak’ females or ‘no peak’ males [9].

### 2.4. FMR1 Methylation Analysis

The *FMR1* methylation analysis was performed using the Methylation Sensitive Quantitative Melt Analysis (MS-QMA) and the EpiTYPER system targeting the Fragile X-Related Epigenetic Element 2 (FREE2), as previously described [10,11]. Briefly, extracted DNA samples were transferred into 96-well plates to be treated with sodium bisulphite. Each DNA sample was bisulphite converted using the EZ DNA Methylation-Gold^TM^ Kit (Zymo Research, Global) in two separate reactions, with each conversion analysed in duplicate reactions using the EpiTYPER system and MS-QMA [10,11].

### 2.5. FMR1 mRNA Analysis

One million peripheral blood mononuclear cells (PBMCs) were isolated per participant from venous blood using Ficoll gradient separation, as previously described [12], with PBMC pellets frozen at −80 °C in RLT buffer (Qiagen Inc., Hilden, Germany) for total RNA extraction. Total RNA was purified using the RNeasy extraction kit, as per the manufacturer’s instructions (Qiagen Inc., Hilden, Germany). A NanoDrop ND-1000 Spectrophotometer (Thermo Fisher Scientific; Global) was used to determine RNA concentrations in triplicate, with purity assessed using the A260/A280 ratio (expected values between 1.8 and 2). Each RNA sample was then diluted to 5 ng/µL, with 2 µL RNA added for complementary DNA (cDNA) synthesis, which was performed using the Multiscribe Reverse Transcription System (Thermo Fisher Scientific; Global) (20 µL total) with 50 units/µL of the reverse transcription enzyme. Real-time quantitative PCR (RT-PCR) was performed on a ViiA™ 7 System (Thermo Fisher Scientific; Global)) to quantify *FMR1*-5′, *FMR1*-3′ and internal control genes (*EIF4A2* and *SDHA*) using the relative standard curve method, as previously described [13].

## 3. Results 

### 3.1. Clinical History 

The patient, a 37-year-old gravida 2 para 1 woman, presented for prenatal diagnosis for FXS. The patient was a known PM carrier (30 and 78 CGG repeats), identified following the diagnosis of her son (proband) with FXS due to mosaicism for both PM and FM alleles. After several failed attempts at in vitro fertilisation with FXS preimplantation genetic diagnosis, the couple achieved a natural pregnancy. A dating scan at 7 weeks and 2 days gestation identified a dichorionic diamniotic twin pregnancy. At 10 weeks gestation, cell-free, non-invasive prenatal testing was performed and demonstrated: (i) a low risk for Trisomy 13, 18 and 21; and (ii) the presence of Y chromosome material (suggesting at least 1 male fetus). CVS was successfully attempted at 12 weeks and 2 days gestation despite obstetric complications and overlapping placentas. Using X and Y specific fluorescence in situ hybridization (FISH) probes, 1 male and 1 female fetus were confirmed.

### 3.2. Initial Diagnostic Testing

**Figure 1:**
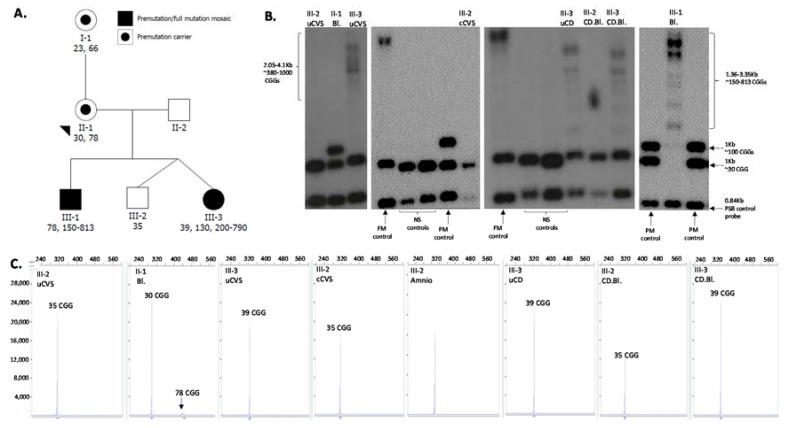
**Pedigree and sizing of the CGG repeat using Southern blot and PCR.** (**A**) Pedigree of the studied family with squares and circles symbolising males and females, respectively. CGG size is indicated by numbers below each participant’s ID (determined on postnatal tissues); (**B**) Southern blot analysis of the *PstI* restriction sites (not methylation sensitive) using the *FMR1* specific and PBS control probes. (**C**) Capillary electrophoresis sizing using standard PCR, as previously described [8]. NS: normal CGG size (<44 repeats); PM: premutation size (55–199 repeats); FM: full mutation (≥200 repeats). The *x* and *y* axes in panel C represent fragment sizes of bp and fluorescence units of capillary electrophoresis profiles from CGG sizing by standard PCR. uCVS: uncultured chorionic villus sampling; Amnio: amniocyte DNA; Bl: blood DNA; cCVS: cultured chorionic villus sampling DNA; uCD: umbilical cord DNA; CD.BL: cord blood DNA.

Southern blot and PCR size *FMR1* testing of uncultured chorionic villi DNA (trophoblasts and mesenchymal cells) showed that Twin 1 (male fetus) had one *FMR1* allele in the normal range of 35 CGG repeats and Twin 2 (female fetus) had one allele of 23 CGG repeats and one allele in the FM range (380–1000 CGG repeats). The patient and her partner were counseled around the spectrum of phenotypes observed among females with FXS. Following a long period of indecision concerning a selective reduction, the couple elected to proceed with the twin pregnancy. Of concern was the observation of the single allele of 35 repeats in Twin 1, an allele size that was inconsistent with the maternal normal allele size of 30 CGG repeats (Figure 1).

This discrepancy may have been due to instability in the normal range or evidence of retraction of a PM or FM present in a small proportion of cells to the normal CGG size allele. To further investigate mosaicism for any expanded alleles in both the male and female fetuses, the uncultured chorionic villus (uCV) was cultured (cCV) and analysed by both Southern blot and PCR sizing. The results for Twin 1 showed the same normal sized allele (35 CGG) with no evidence of an expanded allele, and Twin 2 had an FM allele (380–1000 CGG).

### 3.3. Follow-Up Prenatal Investigation

**Figure 2:**
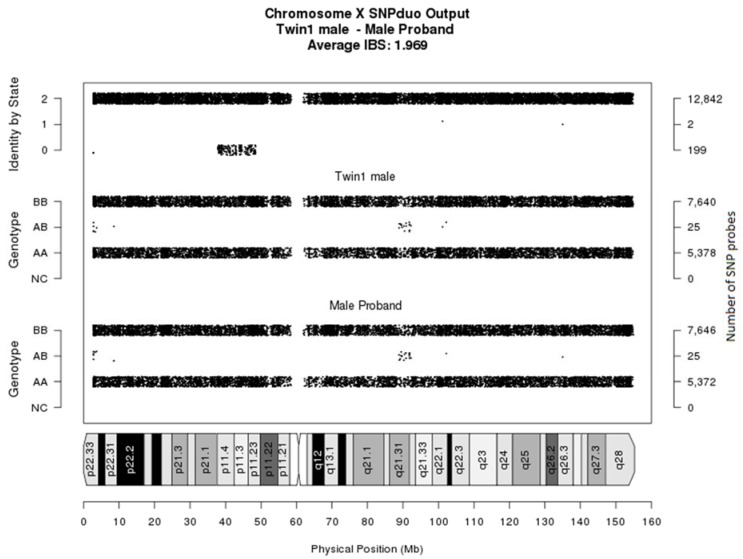
**Chromosome X SNPduo comparison.** A SNPduo comparative analysis of Illumina core exome single-nucleotide polymorphism (SNP) microarray genotyping data was performed using samples from Twin 1 (male) and the male proband. The output analysis showed that the two male siblings share identical alleles (identified by State 2) for chromosome regions Xpter (p11.4) and Xp11.23 (qter), including the *FMR1* gene. This demonstrates the inheritance of the same X chromosome, with the exception of a small region of recombination (identified by State 0) for chromosome region Xp11.4 (p11.23).

As part of further investigations, an Illumina CytoSNP300K microarray (Illumina, Global) was performed on both the male proband (saliva DNA) and Twin 1 (cultured CVS DNA). A comparison of single-nucleotide polymorphisms (SNPs) between the X chromosomes of the affected proband and Twin 1 showed concordance for the majority of the X chromosome SNPs, especially in the *FMR1* region. This confirmed that Twin 1 had inherited the X chromosome with the 35 CGG repeat allele from the mother’s PM chromosome (Figure 2).

Amniocentesis and fetal blood sampling were offered to the patient but declined due to the late gestation. A blood sample was collected from the mother of the PM female (I-1) to investigate the stability of this familial allele; the results demonstrated a typically-behaving smaller PM (66 CGG) repeat size.

### 3.4. PostNatal Investigation

**Figure 3:**
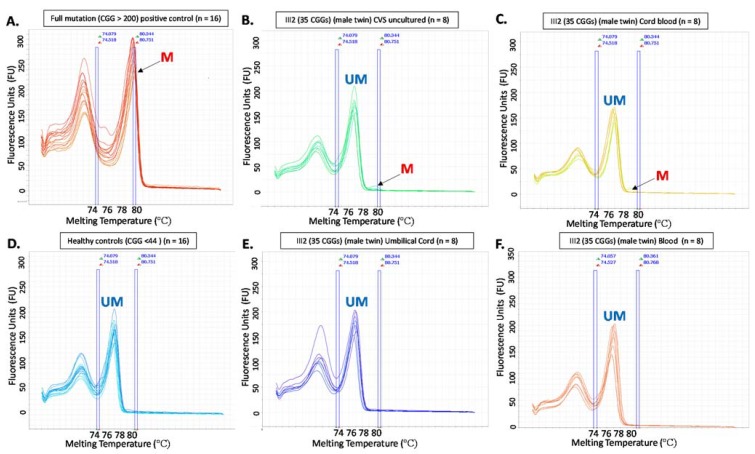
High resolution melt derivative plots from Methylation Sensitive Quantitative Melt Analysis (MS-QMA) analysis of Fragile X-Related Epigenetic Element 2 (FREE2) methylation in the male twin (III-2). Derivative plots in pre and postnatal tissues (**A**–**F**) represent cluster results based on similarities of melt curve placement and shape as determined using the Applied Biosystems^®^ High resolution Melt Software (Thermo Fisher Scientific, Global). Positive and negative controls (**A**) and (**D**) have been included for comparison with the results for the male twin’s samples (**B**, **C**, **E** and **F**), providing details about the presence or absence of the abnormally methylated (M) and unmethylated (UM) *FMR1* alleles. The numbers above each panel represent technical replicates. Note: for each plot X-axis represents melting temperature, while y-axis represents fluorescence released, with each fluorescence unit (FU) equal to 1000 units generated by the Applied Biosystems ^®^ High resolution Melt Software (Thermo Fisher Scientific, Global).

The pregnancy was complicated by preeclampsia and the twins were delivered by caesarean section at 35 weeks gestation. Testing was performed at birth on multiple tissue types by *FMR1* sizing PCR, Southern blot analysis, *FMR1* promoter methylation analysis in multiple tissues and *FMR1* mRNA analysis in blood (Table 1). Twin 1 showed only the 35 CGG allele in all tissues tested, with normal *FMR1* methylation in DNA from cord blood and postnatally-collected venous blood. Reverse transcription real time PCR *FMR1* mRNA studies (blood) showed normal levels. Twin 2 was inconsistent with prenatal results in that mosaicism for a FM and PM was detected on both cord tissue and cord blood DNA, most probably due to postzygotic retraction from the FM range. For comparison, *FMR1* mRNA and methylation results from the older brother affected with FXS (III-1) and the PM mother (II-1) with normal intellectual functioning have been included in Table 1. For both twins, the *FMR1* methylation results were normal in most tissues (consistent with prenatal CGG testing). However, low level methylation mosaicism was detected in the cord blood and uncultured CVS of the male twin (Figure 3).

## 4. Discussion

Intermediate CGG alleles have been shown to expand to FM over two generations, and PM alleles > 55 CGG repeats have been demonstrated to expand to FM in one generation. PM alleles generally increase in size through maternal transmission and very rarely decrease, especially back to the normal range. Furthermore, mosaicism for PM/FM is reported to be present in 12% to 41% of all Fragile X-affected males [15]. Retractions from FM to normal allele size have been reported to be rare by some studies [15], but more common by others [16]. These have also been reported in the prenatal setting [17] without postnatal follow-up. The cause of these retractions is unknown but may be due to excision of the expanded FM or PM CGG repeat in the early postzygotic period, producing mosaic normal size/grey zone size (GZ 45-54 CGGs)/PM/FM results with tissue variability. Most notably, Alfaro et al. reported both expansion and contraction of a maternal PM (93 CGGs) carrier in fraternal twins in the postnatal setting [18]. This report detailed dizygous twins where the male twin demonstrated a FM expansion and the female twin showed a retraction of the mother’s PM allele to the GZ range (54 CGGs).

There is one previous report of FM and GZ alleles being identified during prenatal testing in a female fetus whose mother had one normal (37 repeats) and one PM (111–118 repeats) allele [19]. Long-range PCR performed on a DNA sample derived from amniotic fluid identified three alleles: a normal allele with 29 CGG repeats, a GZ allele with 48 repeats and an FM allele with more than 200 repeats. Further testing identified that the GZ allele had arisen as a result of a postzygotic retraction event in some of the cells, which was confirmed at birth in multiple tissues. There has also been a prenatal report of mosaicism in a male fetus that demonstrated instability/retraction from FM to PM range and placental tissue mosaicism [20]. Another study summarised 26 cases reported in the literature regarding likely retraction and repeat size mosaicism, suggesting that this phenomenon is not uncommon [16].

In this case, the discrepancy in the normal-sized allele difference between Twin 1 and the maternal normal allele size (30 CGG) warranted further investigation. As noted in previous prenatal reports, if inconsistencies in the inheritance of alleles are identified, further investigation including different tissue types should be performed [20]. Twin 1 showed no inconsistency between different tissue types prenatally (or postnatally) indicating there had been either instability of the normal X chromosome allele (30 CGG) or full retraction of the PM maternal allele. The X chromosome SNP linkage comparison with the male pro-band demonstrated that the abnormal X chromosome was inherited by Twin 1, suggesting a retraction may have occurred. Multiple maternal tissues (blood and buccal) showed no mosaicism for a 35 CGG allele, indicating this allele may have arisen early in embryogenesis. One possible explanation for the mechanism could be a deletion from PM to normal (as there were no PM or FM alleles detected in the fetus prenatally or postnatally). This is supported by a previous report that demonstrated four FM patients with deletions of both CGG repeats and flanking *FMR1* coding sequence [21]. Another possibility could be that the mother’s PM expanded to a FM that somatically retracted to normal size early on in embryogenesis.

Whether retraction and/or deletion occurred postzygotically in the fetus, meiotically in the mother, or postzygotically in the mother (maternal germ line mosaicism) is uncertain. An opportunity to test conclusively for maternal germ line mosaicism was negated by the caesarean complications. There is also a possibility that a remote *cis*- or *trans*-acting factor influenced the DNA replication fork at the *FMR1* CGG repeat [22]; this could be examined in future studies to explain the mechanism/s resulting in the repeat instability observed in both twins and their older brother. The etiology of the 35 CGG allele in the male twin is uncertain, despite the *FMR1* mRNA levels being in the normal range. Specifically, while *FMR1* mRNA regulation may not be compromised in blood, this does not rule out potential issues with FMRP translation in blood or other tissues, or small contractions in the flanking sequence that may prevent FMRP from being fully functional. While sequencing of the flanking regions to rule out small deletion/s in close proximity to the repeat was not performed (study limitation), the microarray analysis and FREE2 methylation analysis by both MS-QMA and the EpiTYPER system indicated that the flanking regions located from ~80 bp downstream of the repeat within the body of FMR1 were intact.

Interestingly, the analysis of *FMR1* transcription in the PBMCs of the older PM/FM mosaic brother also revealed an mRNA level within the normal male expression range (Table 1). This is consistent with FREE2 methylation results by both MS-QMA and the EpiTYPER system where approximately half of all cells had a PM or a FM allele with an unmethylated promoter, overexpressing *FMR1* by at least twice the normal level, as previously described [12], but overall, showed normal *FMR1* mRNA levels because the remaining cells had a completely methylated *FMR1* promoter with no detectable *FMR1* mRNA. This postulate is consistent with another study of a PM/FM mosaic male with FXS (harboring a retraction to normal size and microdeletion in the CGG 5’ flanking region), with normal *FMR1* mRNA levels in PBMCs [23]. In contrast to this previous report, Grasso et al. reported four cases of complete retraction to normal range that only had CGG repeat retractions with no flanking coding region deletions [21]. While in one case with FM and normal allele mosaicism, Grasso et al. did demonstrate the presence of FMRP, *FMR1* mRNA levels were not examined.

Although the low level CGG size mosaicism for larger alleles in the male twin was not detected in this study, it may still be present and could be tissue-limited. The MS-QMA analysis did detect low level methylation mosaicism in the cord blood and uncultured CVS of the male twin (Figure 3), which has not been present in any male controls tested to date. This may either represent the presence of a FM allele in a small proportion of cells (that cannot be detected by CGG sizing due to limited analytical sensitivity), or maternal cell contamination where there is partial methylation due to X-inactivation. The cognitive development of Twin 1 with age will most likely give further evidence as to when/where this retraction has occurred, and whether Twin 1 is a non-mosaic carrier of the normal allele.

Twin 2 demonstrated retraction from the full mutation detected on uncultured and cultured CVS to PM size alleles These PM alleles were detected postnatally and are of a different size to the mother’s PM allele, likely produced by postzygotic retraction of the FM allele. The fact that normal methylation in both the uCV, cCV and cord blood was detected, may indicate that in most cells, the FM alleles are on the inactive X-chromosome, otherwise the total methylation detected by MS-QMA would have been above the normal level.

## 5. Conclusions

Whilst a number of cases of contractions into the grey zone or normal range have been previously reported [16,18,21,23], this is the first study to provide postnatal follow-up (repeat sizes, methylation in multiple tissues and *FMR1* mRNA levels in blood) of a normal sized allele in a male twin (with a FM female sibling). Retraction to the normal CGG size in a male twin of a PM mother is an important finding in prenatal diagnosis that should not be underestimated and may be underdiagnosed due to testing regimes. We hypothesise that carriers of retracted normal alleles, if not detected at prenatal diagnosis, may have normal clinical presentation with no recurrence risk. Deletion of the CGG can, however, be associated with clinical abnormality, as demonstrated by Hwang et al. in a case that showed mosaicism for a FM allele and a normal/PM allele with deleted flanking *FMR1* coding regions [23]. Normal alleles that are not consistent with inheritance from parental allele sizes should be investigated by linkage for the abnormal X chromosome and investigated for the presence of normal *FMR1* mRNA and/or FMRP levels. The recurrence risk of expansion and retraction to PM or FM for the mother may be high due to the history of three conceptions demonstrating retractions. This case is also the second report of a twin conception [18] that has demonstrated both expansion and retraction, suggesting a possible association between the twinning process and PM allele instability.

## Figures and Tables

**Table 1:** Summary of *FMR1* testing performed in prenatal and postnatal tissues.

ID	Sample type	CGG size	MS-QMA (MR)	EpiTYPER (MR) CpG10-12 FREE2	*FMR1* mRNA
III-1 (older brother)	Blood	78; 150–813	0.55 (<0.03; n = 23)	0.58 (<0.07; n = 17)	0.98#
III-1 (older brother)	Buccal	78; 150–813	0.54 (<0.03; n = 23)	0.69 (<0.07; n = 17)	-
II-1 (PM mother)	Blood	30; 70	0.21 (<0.37; n = 88)	-	-
II-1 (PM mother)	Buccal	30; 70	0.19 (<0.36; n = 26)	-	-
II-1 (PM mother)	Saliva	30; 70	0.2 (<0.4; n = 33)	-	-
III-2 (male twin)	Uncultured CVS	35	0.01* (<0.03; n = 7)	-	-
III-2 (male twin)	Amnio	35	-	-	-
III-2 (male twin)	Cord blood	35	0.01*(<0.03; n = 23)	-	-
III-2 (male twin)	Umbilical cord	35	0.01	-	-
III-2 (male twin)	Blood	35	0.01 (<0.03; n = 23)	-	1.16#
III-3 (female twin)	Uncultured CVS	39; 380–1000	0.269	-	-
III-3 (female twin)	Umbilical cord	39; 130; 200–790	-	0.35 ( <0.43; n = 154)**	-
III-3 (female twin)	Cord blood	39; 130; 200–790	0.27 (<0.37; n = 88)	0.3 (<0.435; n = 154)**	-

CGG sizes for different alleles are separated by a semicolon; abnormal results are highlighted in bold. MR: methylation ratio. The control maximum methylation values and the number of samples tested are indicated in brackets. * indicates abnormal result from high resolution melt derivative plots presented in Figure 3. ** indicates the EpiTYPER methylation cutoff associated with intellectual disability, as represented by a verbal IQ < 70, established as part of previous studies [14]. (-) indicates that the sample was not available for analysis. To establish the maximum methylation values for (postnatal/prenatal) controls, male and female samples were collected (pre/postnatally) with CGG repeat sizes ranging between 13 and 44 CGGs. No methylation control reference values were available for umbilical cord and uncultured female chorionic villus (CVS) DNA. # The *FMR1* mRNA minimum and maximum values in blood were 0.91 to 1.9 arbitrary units for males, from 30 typically-developing individuals with a CGG size in the normal range < 44 CGGs.
